# Using citizen science to test for acoustic niche partitioning in frogs

**DOI:** 10.1038/s41598-022-06396-0

**Published:** 2022-02-14

**Authors:** Slade Allen-Ankins, Lin Schwarzkopf

**Affiliations:** College of Science and Engineering, James Cook University, Townsville, QLD 4811 Australia

**Keywords:** Ecology, Animal behaviour

## Abstract

The acoustic niche hypothesis proposes that to avoid interference with breeding signals, vocal species should evolve to partition acoustic space, minimising similarity with co-occurring signals. Tests of the acoustic niche hypothesis are typically conducted using a single assemblage, with mixed outcomes, but if the process is evolutionarily important, a pattern of reduced acoustic competition should emerge, on average, over many communities. Using a continental-scale dataset derived from audio recordings collected by citizen scientists, we show that frogs do partition acoustic space. Differences in calls were predominately caused by differences in spectral, rather than temporal, features. Specifically, the 90% frequency bandwidths of observed frog assemblages overlapped less than expected, and there was greater distance between dominant frequencies than expected. To our knowledge, this study is the first to use null models to test for acoustic niche partitioning over a large geographic scale.

## Introduction

Acoustic signaling is important for communication in many animal species. Thus, species are typically surrounded by many interspecific signals^1^, which may interact acoustically, impeding the detection and localisation of conspecific signals^2^, or may lead to signal confusion, causing inappropriate behaviours or lack of responses detrimental to fitness, including reduced mating or accidental matings with heterospecifics^3,4^. To avoid these costs, we expect that animals should employ strategies that reduce acoustic competition^5–7^. This idea has been formalised in the acoustic niche hypothesis, in which acoustic space can be viewed as a niche axis that can be partitioned to avoid negative impacts of co-occurring signals^8,9^. However, clear support that acoustic niche paritioning occurs in nature remains elusive.

Acoustic niche partitioning may occur via evolutionary signal divergence, or behavioural and ecological responses that allow temporal or spatial segregation by species with similar signals^3^. However, studies of acoustic niche partitioning typically examine single species pairs^10,11^, or a single community^5,12–18^. To properly test hypotheses about community ecological processes, the average response of many communities must be compared with a null model assuming no process^19^. To date, however, there have been few attempts for acoustic communities^3^, with mixed results^20–23^. Clearly, a wider examination determining the generality of acoustic niche partitioning in structuring animal communities is needed.

Frogs are an ideal group with which to study acoustic niche partitioning^20^. For most frogs, breeding success relies on females detecting and locating conspecific advertisement calls^24^. Frogs often call for extensive periods, and form choruses in which hundreds of individuals may be vocalizing on the same night^25^. They also rely on similar ecological conditions for breeding (e.g., rainfall) which may limit the ability for temporal and spatial avoidance^26,27^. Additionally, the ability to achieve acoustic niche partitioning through signal divergence may be limited by the strong relationship between call frequency and body size^28^. All of this suggests that competition for acoustic space will be high in frog assemblages, particularly assemblages with many different species, and one would expect partitioning of the acoustic space.

**Figure 1:**
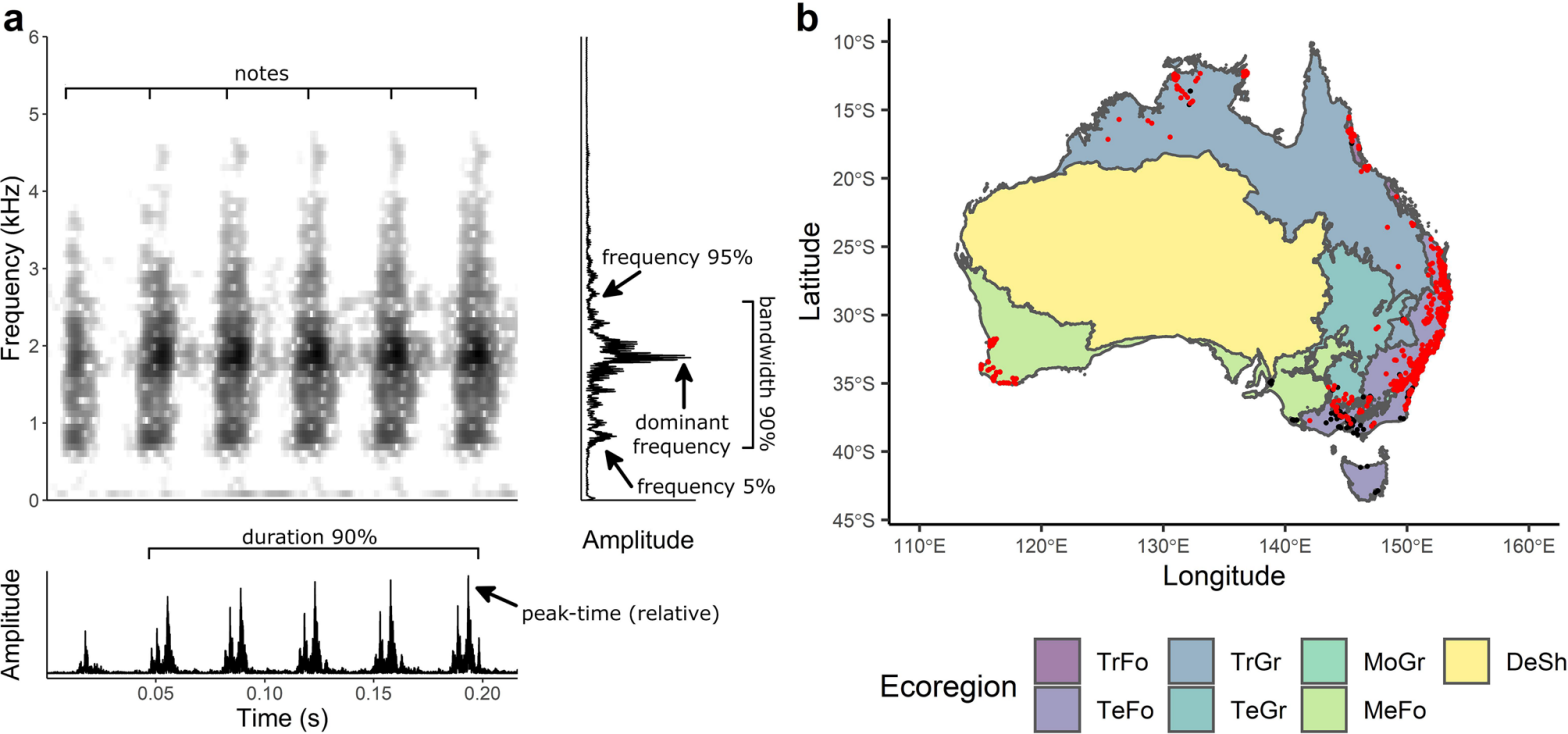
(**a**) Spectrogram, oscillogram and power spectrum of sample frog call (*Adelotus brevis*) showing the various call parameters measured to determine acoustic similarity among calls of different frog species. (**b**) Map of records used for spectral overlap, PCA_All_, PCA_Spectral_ and PCA_Temporal_ (red points, n = 1641), and dominant frequency distance (red and black points, n = 1854) analyses overlayed on Australia’s 7 ecoregions representing the different broad habitat types from which frog assemblages originate (TrFo = Tropical and Subtropical Moist Broaleaf Forests, TeFo = Temperate Broadleaf and Mixed Forests, TrGr = Tropical and Subtropical Grasslands, Savannas and Shrublands, TeGr = Temperatre Grasslands, Savannas and Shrublands, MoGr = Montane Grasslands and Shrublands, MeFo = Mediterranean Forests, Woodlands and Scrub, DeSh = Deserts and Xeric Shrublands). Figures generated using R version 3.6.1 (https://www.r-project.org/). Annotations on a) added with GIMP version 2.10.6 (https://www.gimp.org/).

The aim of this study was to conduct a continent-wide test of the acoustic niche hypothesis using frog assemblages. We used a dataset generated from audio recordings collected by citizen scientists around Australia over one year^29^. This allowed us to examine a much larger number of assemblages than has previously been possible. Using null models, we compared observed to expected levels of interspecific acoustic similarity in these frog assemblages. If the composition of frog assemblages were random with regard to the properties of their calls (i.e. frogs were not partitioning the acoustic space), then we expected no difference in acoustic similarity between observed and random assemblages. However, if frogs were partitioning acoustic space, then we expected lower acoustic similarity among species calls in observed assemblages when compared to random assemblages. To understand which aspects of species’ calls are responsible for any acoustic partitioning, we examined frog assemblages using multiple call features together, as well as spectral and temporal call features separately (Fig. 1).

## Results

We measured the acoustic similarity of observed and random assemblages using five different measures. The first three measures of acoustic similarity used the Euclidean distance (d) between all species pairs using the first three principal components of a PCA fit with (1) six call parameters, (2) three frequency call parameters only, and (3) three temporal call parameters only, hereafter referred to as PCA_All_, PCA_Spectral_, and PCA_Temporal_ respectively. The last two measures of acoustic similarity used specific call frequency variables: (1) spectral overlap (p)—mean proportion of overlap between two species’ 90% log_10_ frequency bandwidths, and (2) dominant frequency distance (log_10_ Hz)—distance between two species’ log_10_ dominant frequencies.

**Figure 2:**
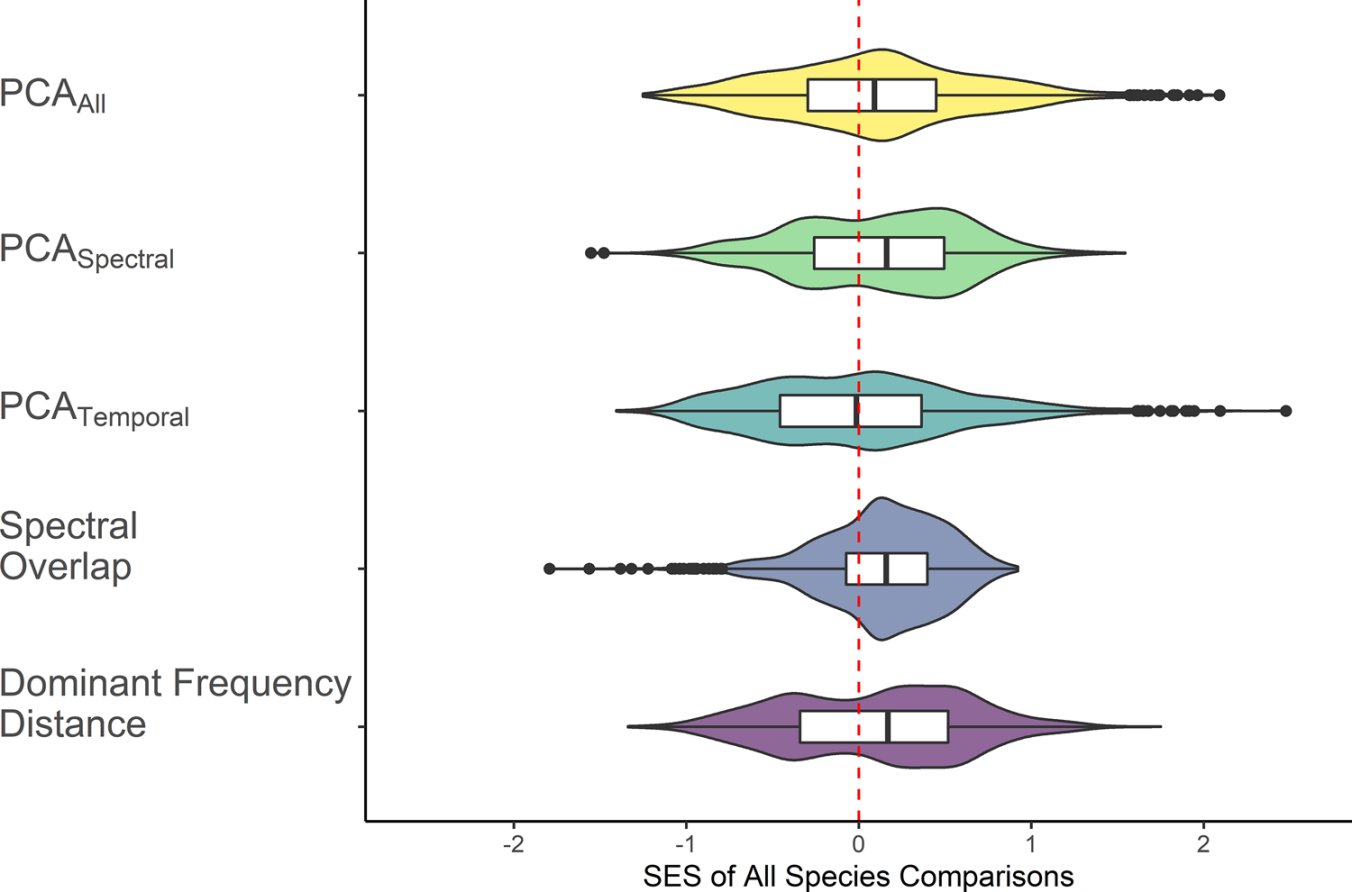
Standardised effect sizes of mean pairwise differences between observed and null assemblages for each measure of acoustic similarity. PCA_X_ measures represent the Euclidean distances between calls from the first three principal components of a PCA on either all measured call parameters, spectral call parameters only, or temporal call parameters only. Spectral overlap is the proportion of overlap between species 90% frequency bandwidths. Dominant frequency distance is the difference between species dominant frequencies in log_10_ Hz. Positive values indicate a trend towards acoustic niche partitioning, while negative values indicate a trend towards acoustic niche aggregation. *Note* The sign of values for spectral overlap have been reversed so that they are comparable with the other four acoustic similarity measures which are all based on distance. Figure generated using R version 3.6.1 (https://www.r-project.org/).

We found evidence of acoustic niche partitioning when comparing observed and expected acoustic similarity for four of the five measures tested (Fig. 2). Standardised effect sizes differed from 0 (mean; 95% CI range; n) for PCA_All_ (0.09; 0.07–0.12; 1641), PCA_Spectral_ (0.12; 0.09–0.14; 1641), spectral overlap (0.14; 0.12–0.16; 1641), and dominant frequency distance (0.10; 0.08–0.13; 1854), but not for PCA_Temporal_ (−0.02; −0.04–0.01; 1641). Of the observed assemblages, 57% were less acoustically similar than the mean of the null assemblages for PCA_All_, 60% for PCA_Spectral_, 70% for spectral overlap, and 59% for dominant frequency.

When accounting for potential covariates using linear mixed-effects models, observed values of PCA_All_, PCA_Spectral_, and dominant frequency distance were still significantly higher (i.e. less acoustically similar) than null values. Similarly, observed values of spectral overlap were still significantly lower (i.e., less acoustically similar) than null values when using the mean of all species’ pairwise comparisons (all p < 0.001). Additionally, both the number of species present in an assemblage and the number of additional species in the surrounding area (i.e. within 50 km) increased the strength of acoustic niche partitioning for each of those measures of acoustic similarity (all p < 0.01).

## Discussion

We provide the first test of the acoustic niche hypothesis, predicting acoustic partitioning, using a large, continental-scale dataset of vocalising frog assemblages. Using a range of call parameters, we found that real assemblages were less acoustically similar than null assemblages. This difference was driven largely by spectral features of calls, rather than temporal features. Specifically, the 90% frequency bandwidths of observed frog assemblages overlapped less than expected, and there was greater distance among dominant frequencies than expected from random distributions. These results support the acoustic niche hypothesis, suggesting that frogs partition the acoustic space to reduce competition.

Most previous studies examining acoustic niche partitioning have focused on a single species pair, or a single community. The studies of acoustic niche partitioning that used null models and multiple or very large communities have come to opposing conclusions. One study examining 11 frog assemblages suggested there was evidence of acoustic partitioning in 3 of those assemblages^20^, however, similar studies involving birds and frogs have reported that species signaling together were more similar than expected by chance^21,22^. Sugai, et al.^22^ and Tobias, et al.^21^ used much longer time scales (1 h and 10 min respectively) than we did (20–60 secs), to determine whether species were signaling together. We found that time-scale is critical to this question, as amphibians partition activity on very fine scales, but tend to aggregate at longer scales, for example aggregating at nightly and seasonal scales^7,27,30,31^. Additionally, the assemblages studied in both Tobias, et al.^21^ and Sugai, et al.^22^ were from small geographic areas compared to our study; perhaps restricted study locations captured a limited range of environmental conditions and were not representative of broad patterns of partitioning of the acoustic space in these animal groups.

There was a significant effect of richness (or the number of species in an assemblage) on acoustic niche partitioning; assemblages with more species showed greater evidence of acoustic niche partitioning. This is consistent with Chek, et al.^20^; in their study the most species-rich frog assemblages showed evidence of acoustic niche partitioning. In our data, variation between observed and null values of acoustic similarity was greater for smaller assemblage sizes (recordings in which fewer frogs were calling). Given the nature of the data collection used in our study (i.e., citizen collected), recordings with fewer species calling may not represent the entire calling community at that location, and could simply be a smaller sub-assemblage calling at a particular moment in time. If this were true, we were likely not always detecting the full extent of acoustic competition at a site.

A number of factors, not accounted for in this study, may cause species’ signals to vary both spatially and temporally^32,33^. It is possible that variation in species calls may either increase or reduce our estimates of the acoustic similarity for each assemblage. Environmental factors, such as ambient temperature, influence species’ call characteristics^34,35^. There may also be regional call variation owing to phylogenetic factors^36^, or species may exhibit signal plasticity, adjusting their signals in response to the presence of other species. Signal plasticity occurs in sympatric and allopatric populations with overlapping species^37–40^, and in response to novel sounds, such as introduced species^41,42^. However, the plasticity is species-specific and does not always reduce acoustic similarity^43^. Despite evidence of plasticity, there are still likely to be constraints on signal variation, because signal recognition is critical to behaviours important to fitness, such as reproduction^44^. Future studies should quantify variations in signals to determine if they further reduce acoustic interference.

Frogs can partition the acoustic space in ways not captured in the recordings used for this study. For example, site-level spatial segregation, where species utilise particular microhabitats within breeding sites, may allow co-occurring species with similar calls to reduce acoustic interference^45^. Whereas some studies have found within-site spatial segregation, and suggested that it may reduce acoustic competition^17,46^, spatial segregation may reflect ecological requirements and not be driven by acoustic avoidance. Fine-scale temporal avoidance, placing calls between those of another species, occurs in birds^7^ and frogs^10,47^, and may also allow co-occurring species with similar calls to reduce acoustic interference. By placing their calls between those of another, species can still co-occur, even with similar signals. Given the frog assemblages used in this study are from audio recordings of 20–60 s duration, it is reasonable to assume that call-level avoidance would be incomplete at best, particularly in assemblages where a large number of species were detected. Both fine-scale spatial and temporal avoidance and the role they play in acoustic niche partitioning in frog assemblages requires further examination.

To our knowledge, this is the first study to examine acoustic niche partitioning in frogs using null models with such a large number of assemblages. Our data show that frog assemblages partition the acoustic space in a way that is consistent with the acoustic niche hypothesis. This supports the results of previous studies that used smaller assemblages, and suggests that acoustic niche partitioning is common in frog communities.

## Methods

### Frog assemblage data

We obtained data on frog assemblages collected by users of the FrogID smartphone app (https://www.frogid.net.au/) from 10 November 2017–9 November 2018^29^. This dataset was developed from 54,864 user-submitted audio recordings (20–60 s duration), uploaded via the app, and checked by FrogID validators to determine all frog species calling^48^. As these data were taken from recordings of 20–60 s, by their nature any species identified in each assemblage were calling at the same time, and potentially competing for acoustic space. Assemblages with less than four species calling, and for which it was not possible to obtain call frequency parameters for all species calling, were removed from the dataset.

### Measuring call parameters and acoustic similarity

Acoustic parameters for each species were measured using high-quality recordings from a range of personal call libraries to ensure call features could be estimated accurately without interference from competing noise sources. Individual calls were isolated and six call parameters were estimated in Raven Pro using a window length of 512 (Table 1, Fig. 1A, Supplementary Table 1; version 1.6, Center for Conservation Bioacoustics): three parameters related to spectral call features (frequency 5%, frequency 95%, and dominant frequency) and three temporal call features (duration 90%, peak-time (relative), and note rate). Notes were defined as subunits of calls that were separated by silence (Supplementary Fig. 1)^49^. All call parameters were estimated from audio recordings stored in the .wav file format and a 44.1 kHz sampling rate. Additional dominant frequency data for species where clear example calls were not available were added from published literature^50,51^. All frequency measurements were log_10_-transformed because aspects of sound perception and production in vertebrates are better described on a ratio scale than a linear scale^52^. Lower (5%) and upper (95%) frequencies were used to characterise the lower and upper frequency bounds of a species call and represent the range of frequencies that may interfere with another species’ call. They were also used to calculate a species’ 90% log_10_ frequency bandwidth. We included dominant frequency, as most authors expect this call parameter to be involved in acoustic niche partitioning^20,28,53^, it is commonly reported, and thus easily obtained for many species. Duration, peak-time (relative), and note-rate call parameters were included to characterise calls based on temporal features, which may also be used by species to distinguish calls and partition the acoustic space (Table 1)^54^.

**Table 1:** Definitions of the three spectral and three temporal call parameters used to measure acoustic similarity between species’ calls. Measurements made using Raven Pro 1.6. Raw frequency measurements were log10-transformed.

Call parameter	Definition
**Spectral**	
Frequency 5% (log_10_ Hz)	Frequency which splits the call at 5% of the total energy of the call
Frequency 95% (log_10_ Hz)	Frequency which splits the call at 95% of the total energy of the call
Dominant frequency (log_10_ Hz)	Frequency with the highest energy
**Temporal**	
Duration 90% (s)	Duration of call containing 90% of call energy
Peak-time (relative)	Relative position in call where peak amplitude occurs (0–1)
Note rate (notes per s)	Number of discrete notes in call divided by the duration of the call

Observed and expected acoustic similarity were determined for each assemblage using five different measures. The first three measures of acoustic similarity used the Euclidean distance (d) between all species pairs using the first three principal components of a PCA fit using: (1) all six call parameters, (2) three frequency call parameters only, and (3) three temporal call parameters only, hereafter referred to as PCA_All_, PCA_Spectral_, and PCA_Temporal_ respectively. All variables were centered and scaled prior to running the PCA (Supplementary Information—Principal Components Analysis). The last two measures of acoustic similarity used specific call frequency variables: (1) spectral overlap (p)—mean proportion of overlap between two species’ 90% log_10_ frequency bandwidths, and 2) dominant frequency distance (log_10_ Hz)—distance between two species’ log_10_ dominant frequencies. This was done for each assemblage using the mean of all species’ pairwise comparisons.

A total of 1641 assemblages (obtained from 20 to 60 s audio recordings) including 73 species were used to measure acoustic similarity using Euclidean distances among principal components (PCA_All_, PCA_Spectral_, PCA_Temporal_), and spectral overlap, while 1854 assemblages including 86 species were used to measure acoustic similarity using dominant frequency distances (Fig. 1B). The number of assemblages differed between analyses because dominant frequency data were available for more species than were all six call parameters. These samples represent ~ 35% and ~ 41% of Australia’s frog species richness, respectively. Assemblage data covered a large temporal and spatial extent, with recordings from 226 (PCA_All_, PCA_Spectral_, PCA_Temporal_, and spectral overlap) and 231 (dominant frequency distance) days of the year, and from five of Australia’s seven ecoregions for PCA_All_, PCA_Spectral_, PCA_Temporal_, and spectral overlap, and six of Australia’s seven ecoregions for dominant frequency distance (Fig. 1B).

### Null model generation and acoustic niche partitioning analysis

A null model approach was used to determine whether observed acoustic niche partitioning was greater than expected by chance. For each assemblage in the dataset, 1,000 random assemblages of matching size were generated using all species present in assemblages within a 50 km radius. Generating random assemblages using only species that occurred in close geographic proximity was necessary, because assemblage structure from species calling in similar habitats are likely to be more similar than random assemblages generated using the entire dataset, which would bias comparisons to suggest real assemblages were similar, but the cause could not be acoustic competition. Assemblages for which there were no other species recorded within a 50 km radius were removed from the data, as observed and expected values would be identical for such cases. Mean values for PCA_All_, PCA_Spectral_, PCA_Temporal_, spectral overlap, and dominant frequency distance were calculated for each of the randomly generated assemblages using the same method as for the observed assemblages.

We searched for evidence of acoustic niche partitioning by calculating the standard effect size (SES) for all assemblages for each measure of acoustic similarity by taking the difference between the observed value and the mean of the 1,000 randomly generated values and dividing it by the standard deviation of the 1,000 randomly generated values. We reversed the sign of SES values for spectral overlap, so that positive values would indicate acoustic niche partitioning, as was the case for the other four measures of acoustic similarity that were based on distance. The mean SES (± 95% CI) for all assemblages for each measure of acoustic similarity was estimated using bias-corrected and accelated bootstrap resampling (n = 10,000). To account for potential covariates, linear mixed effects models were also fit for each measure of acoustic similarity. Observed and null values were compared using the number of species in the assemblage, and the number of extra species within a 50 km radius, as fixed effects, and habitat, month of recording, day of recording and assemblage as random effects. Habitat for each assemblage was classified using the World Wildlife Fund’s ecoregions classification system (http://maps.tnc.org/gis_data.html—accessed 2020-11-13). All analyses were conducted using R version 3.6.1 (R Core Team, 2019), and the lme4 v. 1.1–23;^55^, lmertest v. 3.1–2;^56^, and factoextra v. 1.0.7;^57^ packages.

## Data availability

The frog occurrence dataset is available at https://doi.org/10.15468/wazqft. All other data are available in the main text or the supplementary materials.

## Supplementary Information

Supplementary Information.
