# Supplementary material for: Using citizen science to test for acoustic niche partitioning in frogs
Source: Sci Rep. 2022 Feb 14;12:2447. doi: 10.1038/s41598-022-06396-0 (PMC8844063; doi:10.1038/s41598-022-06396-0)
Supplement: Supplementary file 1 — Supplementary Information. [file 41598_2022_6396_MOESM1_ESM.pdf]

Using citizen science to test for acoustic niche partitioning in frogs

Slade Allen-Ankins[1*], Lin Schwarzkopf[1]

[1] College of Science and Engineering, James Cook University; Townsville, Qld 4811, Australia.

* Corresponding author: Slade Allen-Ankins

Email: slade.allenankins@jcu.edu.au

Supplementary Table 1. Six call parameters used for determining acoustic similarity of observed and null assemblages. Frog call recordings came from the call libraries of the authors, J.Rowley, D.Roberts, Nature Sounds and from published literature. Nature Sounds were from D. Stewart's recordings: Australian Frog Calls Tropical North-east and Australian Frog Calls Subtropical East.

Species	Frequency 5% (Hz)	Frequency 95% (Hz)	Dominant frequency (Hz)	Duration 90% (s)	Peak-time (relative)	Pulse rate (notes per s)	Source
Adelotus brevis	861	2283	1852	0.15	0.9	27.8	J.Rowley
Austrochaperina fryi			3400				Tonini_etal_2020
Cophixalus australis			2950				Tonini_etal_2020
Cophixalus ornatus			2730				Tonini_etal_2020
Crinia bilingua	2842	3962	3618	0.98	0.4	26.4	J.Rowley
Crinia deserticola	3962	4910	4221	0.05	0.7	21.2	Nature Sounds
Crinia georgiana	1809	2756	2498	0.08	0.6	5	D.Roberts
Crinia glauerti	3101	4134	3876	0.38	0.8	15.4	D.Roberts
Crinia insignifera	2584	3359	3187	0.2	0.5	3.2	D.Roberts
Crinia parinsignifera	3704	4651	4307	0.17	0.5	4	J.Rowley
Crinia pseudinsignifera	2584	3790	3359	0.23	0.5	13.1	J.Rowley
Crinia remota	2756	4048	3618	0.68	0.5	15	Nature Sounds
Crinia signifera	2153	2842	2670	0.05	0.6	19.5	J.Rowley
Crinia sloanei			2600				Tonini_etal_2020
Crinia subinsignifera	2498	3359	3187	0.46	0.5	1.3	D.Roberts
Crinia tasmaniensis			2985				Tonini_etal_2020
Crinia tinnula	2929	3618	3273	0.04	0.2	3.5	Nature Sounds
Cyclorana alboguttata	775	2670	1981	0.06	0.4	5.9	Nature Sounds
Cyclorana brevipes	1378	2756	2239	1.13	0.8	0.6	Nature Sounds
Cyclorana novaehollandiae	603	1120	689	0.17	0.5	3.3	Nature Sounds
Cyclorana platycephala			884				Anstis_etal_2016
Cyclorana verrucosa			764				Tonini_etal_2020
Geocrinia laevis			3000				Tonini_etal_2020
Geocrinia leai	2756	3618	3445	0.13	0.1	11	J.Rowley
Limnodynastes convexiusculus	947	2067	1981	0.07	0.5	8.1	Nature Sounds
Limnodynastes dorsalis	345	861	689	0.07	0.2	4.6	D.Roberts
Limnodynastes dumerilii	258	603	345	0.06	0.2	6.9	J.Rowley
Limnodynastes fletcheri	1120	1637	1550	0.09	0.5	5.2	Nature Sounds

Limnodynastes interioris			247				Tonini_etal_2020
Limnodynastes peronii	431	1550	1120	0.03	0.1	8.4	J.Rowley
Limnodynastes salmini	517	1206	603	0.06	0.4	6.1	Nature Sounds
Limnodynastes tasmaniensis	861	2412	1809	0.16	0.9	18.9	J.Rowley
Limnodynastes terraereginae	517	1206	947	0.03	0.1	8.7	J.Rowley
Litoria adelaidensis	1723	3876	3790	0.09	0.5	6	D.Roberts
Litoria aurea	861	1809	1206	1.98	0.7	1.1	Nature Sounds
Litoria barringtonensis	1981	3531	2929	0.64	0.3	3.3	J.Rowley
Litoria bicolor	2498	5771	5168	0.2	0.7	2.6	Nature Sounds
Litoria brevipalmata	1292	3273	2842	0.03	0.4	11.5	J.Rowley
Litoria caerulea	431	1637	1378	0.17	0.7	2.8	J.Rowley
Litoria chloris	1206	2412	1981	0.94	1	0.8	J.Rowley
Litoria citropa	947	1637	1292	0.56	0.9	1.9	J.Rowley
Litoria dentata	2842	4048	3876	0.76	0.6	1	J.Rowley
Litoria ewingii			2286				Tonini_etal_2020
Litoria fallax	4221	5082	4737	0.22	0.8	3.2	J.Rowley
Litoria freycineti	1292	3015	2670	0.08	0.7	6.3	J.Rowley
Litoria gracilenta	1550	3015	2326	1.51	0.3	0.5	J.Rowley
Litoria inermis	3101	3876	3445	0.1	0.8	4.6	Nature Sounds
Litoria infrafrenata	775	2326	1809	0.1	0.6	12.9	Nature Sounds
Litoria jervisiensis	2412	2842	2584	0.46	0.6	1.3	J.Rowley
Litoria latopalmata	1637	3531	3273	0.04	0.7	10.9	J.Rowley
Litoria lesueuri	1120	1637	1378	0.3	0.8	2.6	Nature Sounds
Litoria microbelos	6632	7407	6977	0.12	0.7	5.2	Nature Sounds
Litoria moorei	689	1723	1550	2.14	1	0.3	D.Roberts
Litoria nasuta	2498	3187	2842	0.08	0.5	4.9	Nature Sounds
Litoria nigrofrenata	1464	3273	2929	0.17	0.8	2.7	Nature Sounds
Litoria pallida	1550	4134	3618	0.34	0.9	2.2	Nature Sounds
Litoria pearsoniana	2153	4307	2412	0.34	0.9	4.8	J.Rowley
Litoria peronii	1034	1895	1723	1.27	0.6	14.2	J.Rowley
Litoria phyllochroa	2067	3359	2929	0.77	0.3	4.3	J.Rowley
Litoria raniformis			1119				Tonini_etal_2020
Litoria revelata	3790	4565	4221	0.12	0.8	5.5	Nature Sounds
Litoria rothii	1120	2067	1809	0.63	0.5	10.2	Nature Sounds

Litoria rubella	2153	3359	2842	0.37	0.8	2.2	Nature Sounds
Litoria tornieri	1809	4221	1981	0.06	0.4	10.1	J.Rowley
Litoria tyleri	1206	3015	2412	0.83	0.6	6.3	Nature Sounds
Litoria verreauxii	2239	2670	2498	0.25	0.9	2.9	J.Rowley
Litoria watjulumensis	1206	3531	3015	0.35	0.9	10.3	Authors
Litoria wilcoxii	861	1206	1034	0.2	0.2	30.1	J.Rowley
Litoria xanthomera	947	2584	2498	0.78	0.9	0.8	Nature Sounds
Mixophyes coggeri			562				Tonini_etal_2020
Mixophyes fasciolatus	689	1292	1120	0.15	0.6	3.5	J.Rowley
Mixophyes iteratus	431	1206	517	0.12	0.7	3.5	Nature Sounds
Neobatrachus pictus			1300				Tonini_etal_2020
Notaden melanoscaphus			447				Tonini_etal_2020
Paracrinia haswelli	2153	2498	2326	0.12	0.6	3	J.Rowley
Platyplectrum ornatum	431	2067	1895	0.03	0.1	9.5	Nature Sounds
Pseudophryne bibronii	1981	3101	2842	0.17	0.5	3.1	J.Rowley
Pseudophryne coriacea	1895	3445	2239	0.13	0.7	4	J.Rowley
Rhinella marina	517	775	603	4.82	0.3	15.9	Nature Sounds
Uperoleia altissima	2498	3359	3101	0.02	0.4	38.3	Nature Sounds
Uperoleia aspera			2778				Tonini_etal_2020
Uperoleia crassa			2356				Tonini_etal_2020
Uperoleia fusca	1895	2670	2412	0.2	0.6	2.6	J.Rowley
Uperoleia inundata	1688	2813	2625	0.1	0.5	63.2	Authors
Uperoleia laevigata	2153	2670	2498	0.16	0.7	3.2	J.Rowley
Uperoleia lithomoda	1809	2842	2584	0.01	0.3	15.8	Nature Sounds
Uperoleia littlejohni	1378	2153	1981	0.02	0.5	13.6	Nature Sounds
Uperoleia mimula	2239	2756	2498	0.06	0.3	38.3	Nature Sounds
Uperoleia minima			3517				Tonini_etal_2020
Uperoleia rugosa	2326	2929	2670	0.07	0.5	32.6	J.Rowley
Uperoleia tyleri	1809	2584	2412	0.24	0.5	2.3	J.Rowley

*Tonini, J. F. R., Provete, D. B., Maciel, N. M., Morais, A. R., Goutte, S., Toledo, L. F., & Pyron, R. A. (2020). Allometric escape from acoustic constraints is rare for frog calls. Ecology and Evolution, 10(8), 3686-3695.

**Anstis, M., Price, L. C., Roberts, J. D., Catalano, S. R., Hines, H. B., Doughty, P., & Donnellan, S. C. (2016). Revision of the water-holding frogs, Cyclorana platycephala (Anura: Hylidae), from arid Australia, including a description of a new species. Zootaxa, 4126(4), 451-479.

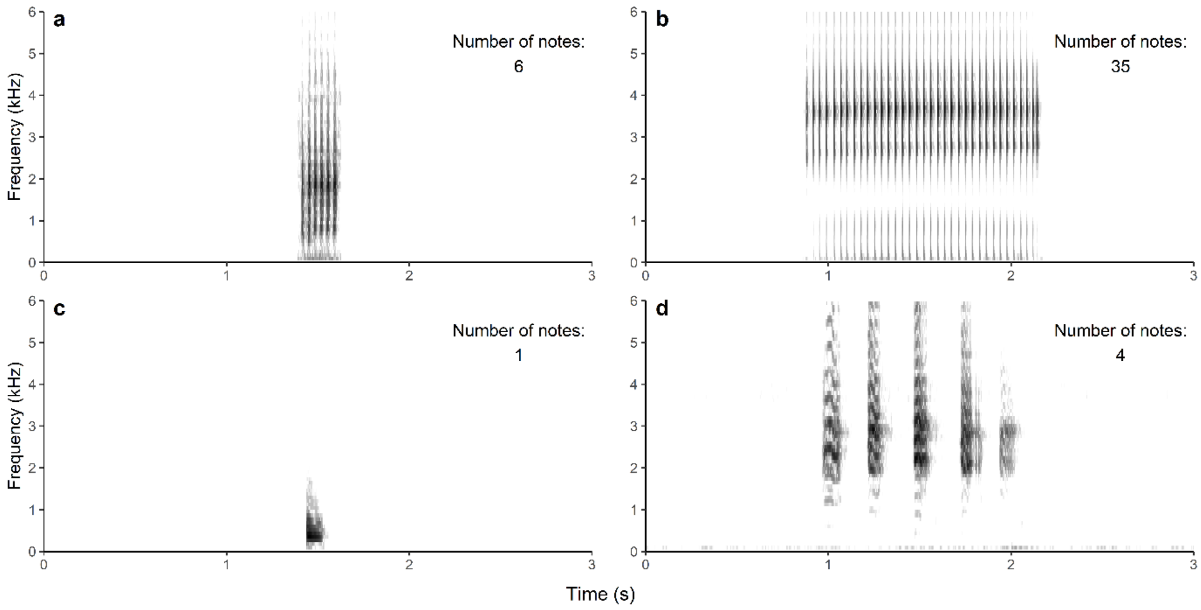

Fig. S1. Example spectrograms of single calls for a) *Adelotus brevis*, b) *Crinia bilingua*, c) *Limnodynastes dumerilii* and d) *Litoria phyllochroa*. The number of individual notes in each call are labelled. Notes were defined as subunits of calls that were separated by silence as per Koehler et al. 2017. Calls provided by J. Rowley. Figures generated using R version 3.6.1 (https://www.r-project.org/).

Principal Components Analysis

The acoustic similarity of calls of all species in the dataset were estimated using three different PCAs run on all six measured call parameters, the three spectral call parameters, and the three temporal call parameters. For the PCA examining all call parameters, the first 3 principal components accounted for ~86% of the variance, with the first PC positively correlated with all three frequency parameters (frequency 5%, frequency 95%, and dominant frequency), the second PC positively correlated with peak-time relative and duration 90% and negatively correlated with pulse rate, and the third PC positively correlated with duration 90% and pulse rate (Fig. S2A). For the PCA examining spectral call parameters, the first 3 principal components accounted for 100% of the variance, with the first PC positively correlated with all three frequency parameters (frequency 5%, frequency 95%, and dominant frequency), the second PC negatively correlated with frequency 5%, and the third PC having low correlation values with all variables (Fig. S2B). For the PCA examining temporal call parameters, the first 3 principal components accounted for 100% of the variance, with the first PC positively correlated with peak-time relative and duration 90%, the second PC positively correlated with duration 90% and negatively correlated with peak-time relative, and the third PC positively correlated with peak-time relative and pulse rate (Fig. S2C).

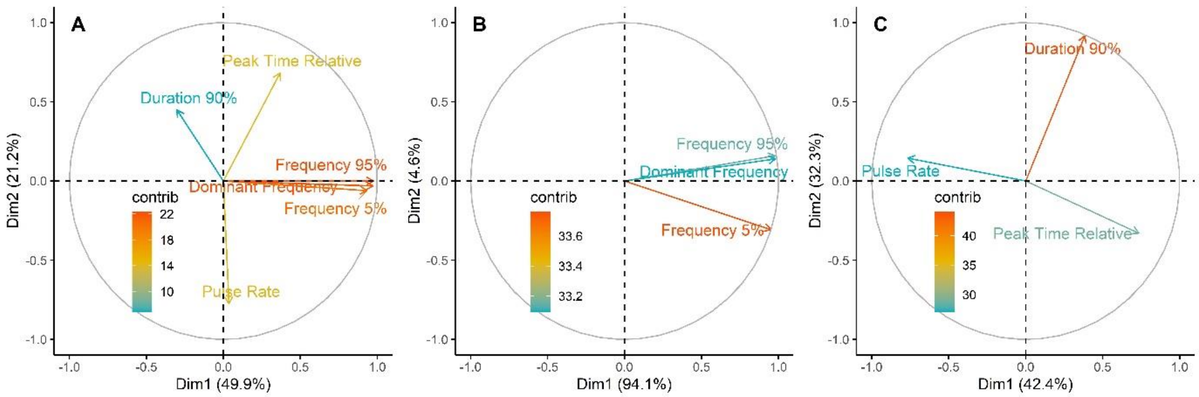

Fig. S2. PCA varplots of A) all six call parameters, B) frequency call parameters only, and C) temporal call parameters only. Figures generated using R version 3.6.1 (https://www.r-project.org/).
